# Bills to Regulate the Practice of Dentistry in Maryland and District of Columbia

**Published:** 1880-02

**Authors:** 


### EDITORIAL, ETC.

Elsewhere is published an abstract of the bill before Congress for the regulation of the practice of Dentistry in the District of Columbia. As might be expected, quite an outcry is made by those whose supposed interests it trenches upon, and it has been found necessary to furnish to Congress the following brief:

"*An act for the regulation of the practice of dentistry in the District of Columbia, and for the protection of the people of said District.*"

An explanation of the objects of this bill, as contained in its title, and a brief statement of the present condition and circumstances of dentistry in this and some other jurisdictions, will furnish the best reasons and arguments why the bill should pass.

The second object named in the title, being the more important, will be first considered.

It is the protection of the people of the District of Columbia against improper treatment and injury at the hands of persons who pretend to practice dentistry, who are ignorant, and consequently unskillful and incompetent, having never been properly educated in any way in this special calling. It may be said that the people have the option to go to a competent or incompetent dentist, and it is their own fault if they choose the latter. But the people, as a general rule, are not qualified to judge between the two, and it is only after they have suffered irreparable injury at the hands of a dentist (and there are many cases where this has occurred) that they can say that he is unskillful and incompetent. Even Senators themselves are not exceptions to this rule, and one at least of their number can be named who has had this sad experience.

Results like this are inevitable under the present condition and circumstances of dentistry in this and some other parts of the country, where any one can practice this calling who chooses to open an office and put up a sign, even without a particle of instruction or experience.

Justice to the people of this District requires that this state of affairs should not be allowed to continue. In many of the States the Legislatures have enacted laws similar to the present proposed bill, and almost every year others are doing the same. This is a necessity that has arisen from the above-named facts, the progress of the age, and the rapid advance of dentistry to a place among the learned professions.

The other object of the proposed law is a natural consequence of this advance. No one can practice law unless regularly admitted to the bar through a diploma or examination, or both. Congress has, by enactment of a law to that effect, said that no one can practice medicine here without a diploma from a medical college and a license from the medical Society of the District of Columbia. Dentistry, like theology, law, and medicine, has its colleges in various parts of the country, that give thorough instruction in the art and science of dentistry, and confer degrees and grant regular diplomas to those pupils who are qualified to receive them. In these institutions—in its high standard of education and learning, in its many organizations, engaged in scientific as well as practical investigations, and in the known results of these labors, it has all the attributes and requirements of a learned profession.

This fact being established, it is but just and proper that it should be recognized as such by the passage of laws that will place it practically, as to its duties and privileges, on the same footing as the other professions, and thus elevate, utilize, and concentrate the efforts of its best members in the proper treatment of that most important part of the human organism—the mouth and teeth.

As to the operation of the law (if passed) upon those practicing dentistry in the District of Columbia, that can be stated in a few words :

1st. No one can commence the practice of dentistry here after the passage of the act who is not in possession of a diploma as evidence of previous instruction and qualification.

2d. No one *now* engaged in the practice of dentistry in this District will be disturbed until April 1st, 1881. Those thus engaged without a diploma will thereby have the time and opportunity to obtain one from some one of the dental colleges, all of which have their sessions or terms during the winter, and ending March 1st of each year, or to prepare themselves for the examination prescribed in this act; and even after April 1st, 1881, this law will affect only those who cannot obtain such diploma or pass this examination.

From this class of incapables only can opposition to this measure proceed.

J. B. Ten Eyck,

R. B. Donaldson,

R. Finley Hunt,

T. O. Hills,

H. B. Noble,

J. Curtiss Smithe,

*Committee.*

Already in Massachusetts, New York, Pennsylvania, Ohio, Michigan, South Carolina, Georgia, and perhaps some other States, laws of similar character have been enacted, and bills are pending before the Maryland, Missouri, and California Legislatures to properly regulate the practice of dentistry and adequately protect not only the public from impostors and incompetents, and protect those who having spent years in the careful acquirement of a proper knowledge of the science and art of dentistry, find themselves injured by those who "pick it up."

H.

---

The following abstract of the Bill to incorporate the Dental Association of Maryland and the District of Columbia, and to regulate the practice of dentistry in this State, is taken from the Balto. *Sun* of Jan. 23d: Under the provisions of the bill "it shall be unlawful for any person or persons to engage in the practice of dentistry in the State for compensation unless said person or persons shall have received a diploma from the faculty of some dental college, duly incorporated under the laws of this or some other State or foreign government, in which is annually delivered, in good faith, a full course of lectures and instructions in dentistry, and in addition thereto shall have obtained a license from a board of dentists, duly authorized and appointed by this Bill to issue such license. The bill exempts from its provisions those persons now engaged in the practice of dentistry in this State. It provides that it is the duty of the Dental Association at its next annual meeting to elect a board of examiners, to consist of five members. to be known as the board of dental examiners in the State. They shall be elected for terms of one, two, three, four and five years respectively, and to fill all vacancies successively for a term of five years. The said board shall elect a president and secretary, and shall meet in Baltimore annually by giving thirty days' notice in the newspapers published at Baltimore, Cumberland and Easton. They are empowered to grant a license to any applicant who shall furnish satisfactory evidence of having graduated and received a diploma from any incorporated dental college of good standing, and who shall undergo a satisfactory examination. It is provided that any person who shall, in violation of this bill, practice dentistry in the State of Maryland for a fee or reward, be liable to indictment in the county or city of Baltimore in which the offense is committed, and upon conviction shall be subject to a fine of not less than fifty nor more than two hundred dollars for each offense. Nothing in the bill shall be construed to prevent physicians or surgeons from extracting teeth or prescribing for diseases of the mouth. Within sixty days after the passage of the bill all persons practicing dentistry within the State are required to register in the office of the clerk of the county where located, in a book to be kept for the purpose, giving his name, office and postoffice address, and the date of such registration, and shall be entitled to a certificate of such registration upon payment to the clerk of a fee of fifty cents. All fines collected under the bill are to go one-half to the Dental Association of Maryland and the District of Columbia and the other half to be given to the common school fund in each county where the offense is committed. The board of examiners are authorized to charge $20 for each license issued to those who desire to practice dentistry. The bill is to take effect from the date of its passage."

